# Chronic neck pain and anxiety-depression: prevalence and associated risk factors

**DOI:** 10.11604/pamj.2016.24.89.8831

**Published:** 2016-05-27

**Authors:** Imane Elbinoune, Bouchra Amine, Siham Shyen, Sanae Gueddari, Redouane Abouqal, Najia Hajjaj-Hassouni

**Affiliations:** 1 Rheumatology Departement, El Ayachi Hospital, Ibn Sina University hospital, Sale, Morocco; 2 Laboratory of Biostatistics, Clinical and Epidemiological Research (LBRCE), Faculty of Medicine and Pharmacy, Ibn Sina University Hospital, Rabat, Morocco; 3 LIPROS-URAC30, Mohammed V Souissi University, Rabat, Morocco

**Keywords:** Chronic neck pain, anxiety, depression, HAD

## Abstract

**Introduction:**

Chronic pain in rheumatology often has a psychic impact, which may aggravate the daily life of patients. Chronic neck pain, as an example, is a frequent reason for consultation. The aim of this study is to assess the prevalence of anxiety and depression in patients with neck pain, and identify risk factors associated with their occurrence.

**Methods:**

It was a cross-sectional study that concerned 80 patients with neck pain lasting for more than 3 months, seen in rheumatology consultations. All patients with symptomatic neck pain or psychological history or receiving psychotropic medication were excluded from the study. For each patient, we determined the sociodemographic characteristics and clinical ones. The anxious and depressed mood was assessed by the Hospital Anxiety and Depression Scale (HAD).

**Results:**

Of the 80 patients, 67 (83.8%) were women. Average age of our population was 51.8± 11.8 years. Median duration of symptoms was 24 months [12, 48]. Mean VAS pain was 63.9% ± 12.5, mean VAS functional discomfort was 60.9% ± 14.2 and mean VAS disability was 59.8% ± 14.7. 32 patients (40%) were illiterate and 18 (22.5%) had university level. Anxiety was found in 54 (68.4%) and 44 (55.7%) patients were depressed. In univariate analysis, VAS disability was statistically linked to anxiety (OR:1.05; 95%CI: 1.01-1.08; p = 0.02). The cervicobrachial neuralgia (CBN) was significantly associated with depression (OR: 3.33; 95%CI: 1.20-9.23; p = 0.02). Primary education level had a statistically significant relationship with anxiety (OR: 6.00; 95%CI: 1.03-34.84; p = 0.04) and depression (OR: 5.00; 95%CI: 1.09-22.82; p = 0.03). In multivariate analysis, VAS disability and CBN were independently associated with anxiety and depression respectively.

**Conclusion:**

This study underlines the fact that anxiety and depression are prevalent in chronic neck pain (CNP) patients. Furthermore, disability and CBN which are linked to CNP can predict which patient is at higher risk of psychological distress.

## Introduction

Chronic neck pain is a frequent reason for consultation. It's a highly prevalent condition with about two thirds of the adult population affected at some time in their lives. Unspecific neck pain usually resolves within days, but in 10% neck pain recurs or persists with algofunctional consequences which are responsible of physical disability and high health care cost [1]. Chronic pain is an unpleasant sensory and emotional experience that may affect negatively the behavior and the well being of the individual, and have a major impact in his family and professional life. Otherwise, neck pain, as any chronic pain, can have a psychological impact which makes daily life difficult and sustains the pathology.

The management of the chronic neck pain is based on different therapeutic options such as exercise, manipulation and mobilization, acupuncture, medicinal and injection therapies. Thus, the assessment of psychological factors such as anxiety and depression can be a complementary approach in the treatment of neck pain and physical disability. Our purpose will be to assess the prevalence of anxiety and depression in a Moroccan population of patients with neck pain, and identify risk factors associated with those both psychological parameters.

## Methods

### Study design

It was a cross-sectional study conducted in Rheumatology Department. Patients received clear informations about the purpose of this survey and replied, during a consultation for chronic neck pain which had common characteristics, to a questionnaire containing different items: sociodemographic, clinical, paraclinical and psychological.

### Recruitement of patients

The study concerned patients over 18 years suffering from neck pain for more than three months and seen during the consultations of Rheumatology. All patients were asked to exclude, from a list of potentially eligible persons, who had their neck pain consultation because of a new trauma, suffered from secondary neck pain (rheumatic, infectious, neoplastic, metabolic) or from psychic disease, received psychotropic drugs or had severe cognitive impairment. Thus, the survey included eighty persons with common neck pain.

### Baseline variables

Sociodemographic items were assessed by single item question: age, gender, body mass index, profession, working hours, education level, marital status, monthly income, exercise practice and different aspects of perceived social support (emotional, instrumental, social). For clinical items, single item questions were used to ask for: disease duration, delay before the first consultation in months, assessment of pain intensity, functional disability and global discomfort using a visual analogue scale, and presence or absence of paravertebral contracture, shoulder pain, cervicobrachial neuralgia, and low back pain.

### Questionnaire

Symptoms of anxiety and depression were assessed using the HADS (Hospital Anxiety and Depression Scale). This self-screening questionnaire was developed by Zigmond and Snaith for detecting and classifying the severity of anxiety and depression [2]. In this study, we used the Arabic version of HADS validated by El-Rufai and Absood [3]. The HADS contains 14 items and consists of 2 subscales: anxiety and depression. Each item is rated on a four-point scale, with 7 items evaluating depression and 7 items assessing anxiety, giving maximum scores of 21 for anxiety (HAD-A) and for depression (HAD-D). According to the German test manual, patients with depression score > 8 were considered depressive, patients with an anxiety score > 10 were considered anxious [4].

### Statistical analysis

All statistical calculations were done by computer using SPSS software (SPSS, Inc., Chicago, IL, USA). Quantitative variables were expressed as means ± SD or as medians and interquartile range, depending on their distribution. For categorical variables, the percentages of patients in each category were calculated. Binary logistic regression was used to identify risk factors in univariate and multivariate analysis. Each variable with a p < 0.10 in univariate analysis was included in multivariate model. Results were reported with odds ratio (OR) and 95% confidence interval (CI). Tests were 2-tailed, with significance set at a p value of less than 0.05.

## Results

Of the 80 neck pain recruited patients, 67 (83.8%) were women. The average age of our population was 51.8 ± 11.8 years (25,74). The median duration of symptoms was 24 months [12, 48]. The mean VAS pain was 63.9 ± 12.55%, the mean VAS global discomfort was 60.9 ± 14.2% and the mean of the VAS functional disability was 59.8% ± 14.7. Thirty two (40%) patients were illiterate and 18 (22.55%) had a university level. Celibacy was noted in 6 (7.55%) neck pain patients. The number of active patients was 65 (81.3%) with an average working hours per day assessed at 7.5 hours ± 1.9. Thirty patients (37.55%) had a monthly income >5000 Dirhams (DH). One third of patients had approximately financial, physical and psychic support.

**Figure 1:**
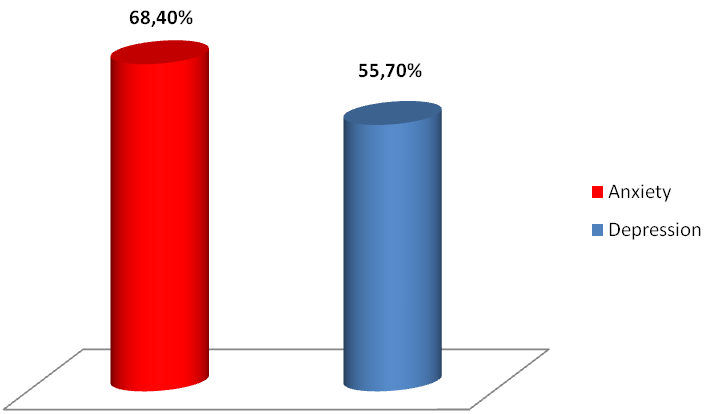
Prevalence of anxiety and depression in neck pain patients

Table 1 reveals the sociodemographic and clinical characteristics of our population. A state of anxiety was found in 54 (68,4%) patients, and 44 (55,7%) from eithy patients had depression. Figure 1 shows the prevalence of anxiety and depression in our sample. In univariate analysis, VAS disability was statistically linked to anxiety (OR:1.05 95% CI: 1.01-1.08 p = 0.02). The cervicobrachial neuralgia (CBN) was significantly associated with depression (OR:3.33 95%CI:1.20-9.23 p = 0.02). A low level of education had a statistically significant relationship with anxiety (OR:6.00 95%CI:1.03-34.84 p = 0.04) and depression (OR:5.00 95%CI:1.09-22.82 p = 0.03). However, there was no significant association between age, gender, duration of symptoms or pain intensity (VAS pain) and HAD-A or HAD-D. In multivariate analysis, VAS disability and NCB were independently associated with anxiety and depression respectively. Table 2 examines the association between different items and anxiety-depression

**Table 1:** Sociodemographic and clinical characteristics of patients

Characteristics	Patients N=80
Age1 (years)	51.8 ± 11.8
Female gender^3^	67 (83.8)
**Education level^3^**	
Illiterate	32 (40)
Primary Secondary	14 (17.7)
University	18 (22.5)
**Marital status^3^**	
Single	6 (7.5)
Married	66 (82.5)
Working patients^3^	65 (81.3)
Working hours per week^1^	7.5 ± 1.9
Exercise practice^3^	15 (18.8)
**Support^3^**	
Financial	34 (42.5)
Physical	28 (35)
Psychic	35 (43.8)
**Monthly income^3^**	
Without	2 (2.5)
<2000 DH	25 (31.3)
2000-5000 DH	23 (28.8)
>5000 DH	30 (37.5)
Duration of symptoms (months)^2^	24 [12; 48]
Delay before the 1st consultation (months)^2^	5 [2; 12]
VAS pain1 (0-100%)	63.9 ± 12.5
VAS global discomfort1 (0-100%)	60.9 ± 14.2
EVA functional disability1 (0-100%)	59.8 ± 14.7
Para vertebrale contracture3	55 (68.8)
Shoulder pain^3^	61 (76.3)
Cervicobrachial neuralgia^3^	27 (33.8)
Low back pain^3^	39 (48.8)

1 mean ± SD /

2 median [IQR] /

3 effective (percentage)

**Table 2:** Factors associated with anxiety and depression

	Univariate analysis						Multivariate analysis					
		HAD-A			HAD-D			HAD-A			HAD-D	
Characteristics	OR	95% CI	p	OR	95% CI	p	OR	95% CI	p	OR	95% CI	p
Age (per year)	1.02	0.98-1.06	0.26	0.98	0.94-1.02	0.28						
Gender												
Female	Ref			Ref								
Male	1.67	0.42-6.68	0.4	0.63	0.19-2.08	0.45						
**Education leve**l												
Illiterate	2.44	0.73-8.17	0.15	2.77	0.82-9.31	0,1	1.56	0.39-6.16	0.52	2.76	0.70-10.90	0.15
Primary	6.00	1.03-34.84	0.04	5.00	1.09-22.82	0,03	4.82	0.71-32.74	0.11	4.58	0.88-23.89	0.07
Secondary	2.20	0.54-8.96	0.27	3.33	0.82-13.64	0,09	1.27	0.26-6.15	0.76	3.66	0.74-18.27	0.11
University	Ref			Ref			Ref			Ref		
**Marital status**												
Single	Ref			Ref								
Married	4.84	0.82-28.70	0.08	8.00	0.88-72.52	0.06	6.92	0.76-63.13	0.08	8.75	0.67-112.9	0.09
**Profession**												
None	Ref			Ref								
Yes	0.53	0.14-2.11	0.37	0.93	0.29-2.98	0.90						
Working hours per week (per hour)	1.10	0.85-1.44	0.46	1.12	0.87-1.45	0,4						
**Exercise practice**												
None	Ref			Ref								
Yes	1.34	0.38-4.72	0.65	0,89	0.28-2.75	0.34						
Duration of symptoms (per month)	1.01	0.99-1.03	0.25	0.99	0.98-1.01	0.84						
Delay before the 1^st^ consultation (per month)	1.01	0.97-1.05	0.48	0.99	0.96-1.03	0.72						
VAS pain (per 1%)	1.02	0.98-1.05	0.46	1.02	0.98-1.06	0.30						
VAS global discomfort (per 1%)	1.03	0.99-1.06	0.14	1.02	0.98-1.05	0.26						
VAS functional disability (per 1%)	1.05	1.01-1.08	0.02	1.01	0.97-1.04	0.63	1.05	1.01-1.10	0.01			
**Paravertebral contracture**												
None	Ref			Ref								
Yes	0.67	0.23-1.97	0.46	0.69	0.26-1.86	0.47						
**Shoulder pain**												
None	Ref			Ref								
Yes	0.99	0.33-3.02	0.99	1.18	0.42-3.31	0.76						
**Cervicobrachial neuralgia**												
None	Ref			Ref						Ref		
Yes	2.01	0.69-5.86	0.19	3.33	1.20-9.23	0.02				4.88	1.41-16.92	0.01
**Low back pain**												
None	Ref			Ref								
Yes	1.27	0.49-3.30	0.62	1.80	0.73-4.43	0.20						

OR: Odds ratio, CI: Confidence interval, p: Value of significance, Ref: Reference category

## Discussion

In our study which concerned eighty patients with chronic neck pain, a state of anxiety was found in 54 (68.4%) cases, and 44 (55.7%) patients had depression. To our knowledge, there are no studies assessing the prevalence of anxiety and depressive disorders in chronic neck pain patients. The majority of studies focusing on neck pain are interested in the relationship between it and psychosocial distress. Recent conceptions of spinal pain, especially chronic back pain, have highlighted the role of psychosocial factors. For instance, Sagheer MA and al reveal in a study an abnormal level of anxiety and depression which is found in 77 (55%) and 68 (48.57%) patients with chronic low back pain respectively [5]. This result concurs approximately with what was found in our sample concerning neck pain.

Persons with chronic pain are more likely to have depressive symptoms than those without pain [6]. Thirty-one at 100% of patients with chronic pain report a diagnosis of depression [7]. Besides, among the psychological factors related to pain, anxiety is the most prevalent factor [8]. People who have persistent pain may feel anxious about the meaning of their symptoms and the future of their pain. Researchers have shown that anxiety about health is higher among people with pain than in the group control [9]. Furthermore, the consensus conference of Anaes concerning management of chronic pain has reported that this physical and emotional experience reproduces anxiety [10].

Regarding the relationship between neck pain and mood distress, in cross-sectional surveys, pain in this location is associated with a psychological distress such as anxiety and depression [11–13]. Psychosocial factors are known risk factors for neck pain [14]. Many studies have demonstrated the importance of psychological factors in the management of chronic pain. Moreover, a prospective cohort study in France shows that psychological factors, among others, are important predictors of incidence or persistence of neck pain [15].

What is more, a recent study of Eva Blozik and al suggests depression and anxiety being major determinants of neck pain [16]. However, Marloes and al shows that pain at the neck among others locations can be a risk indicator of depression and/or anxiety disorders onset [17].

Our survey evaluated the mood of patients at the moment of the consultation, and suggested that individuals with chronic neck pain were at high risk to experience anxiety and depression. However, we cannot conclude that the psychological distress is risk factor or consequence of pain.

Assessing different chronic neck pain characteristics in relation to depressive and anxiety disorders plays an important role in providing information for practitioners regarding which characteristics or variables are associated with psychological distress in patients suffering from neck pain. In current neck pain population, young age was not related to psychological distress which could be associated with neck pain. Also, be a woman was not a risk factor of being anxious or depressed. The insignificant association between marital status, professional occupation and anxiety-depression in patients with neck pain suggested that family responsibilities, and stress at work or professional factors were not related to anxiety-depression in our population. Also, lack of practicing exercise was not linked to psychological parameters associated to pain in this location.

However, the present study showed that subjects with a primary education level (17.5%) had a higher chance of being anxious (OR:6.00 95%CI:1.03-34.84 p = 0.04) or depressed (OR:5.00 95%CI:1.09-22.82 p = 0.03) than those with high level.

Concerning clinical variables, duration of evolution's symptoms and delay before the first consultation were not associated nor with anxiety nor with depression. Furthermore, pain intensity and global discomfort which were assessed by visual analogue scale were not associated nor with HAD-A nor with HAD-D. This may mean that pain intensity and global discomfort don't predict which patients with chronic neck pain will be more anxious or depressed. Yet, functional disability was related to being anxious (OR:1.05 95%CI:1.01-1.08 p = 0.02). Thus, the disability which can be secondary to pain makes the patient feel anxious about his future.

In this survey, neck pain accompanied by paravertebral contracture, shoulder pain or low back pain was not more purveyor of altered psychic state. But, a cervicobrachial neuralgia (CBN) could be a predictor factor (OR:3.33 95%CI:1.20-9.23 p = 0.02) of depression. Lack of financial, physical and psychological support was not an added factor to neck pain in order to cause psychological distress.

So, univariate analysis indicated markers for patients with chronic neck pain that could led to anxiety and depression disorders. Primary education level, functional disability, presence of CBN were linked to these both psychological disorders, representing characteristics easy to assess in general practice. Yet, multivariate model suggested functional disability being determinant of anxiety, and CBN being determinant of depression. Thus, education level was confounding factor.

Our findings underline the findings of the literature. Irrespective of being young or old, female or male, pain in different locations including the neck pain seems to be a risk indicator of incident depressive and anxiety disorders. However, intensity of and disability related to pain symptoms, as well as pain in several locations, are associated with an increased likelihood of developing a depressive or anxiety disorders, whereas duration of pain symptoms is not [17]. Also, the study of Eva Blozik and al suggests that deficit in social support, basic education and infrequent exercise are confounding factors and that variability in neck pain levels is intrinsically explained by psychosocial characteristics [16].

Under spinal pain, individuals with low back pain (LBP) who are at high risk to experience anxiety and depression are females [5]. Besides, psychological distress is known significantly linked to LBP which is more prevalent in women and in patients with basic education [18]. Moreover, disability associated to LBP is weakly correlated with psychobehavioural factors [19].

Authors have studied the role of sociodemographic variables such as age, education level, gender and marital status in order to predict which patients with chronic pain are at higher risk of developing depressive or anxious symptoms. The results are significantly different from one study to another. Some studies find no relationship between age and depression in chronic pain [20], while others suggest younger chronic pain patients are most depressed [21]. The same results have been revealed regarding education level [22]. To sum up, depressive comorbidity is found mainly in the older participants, women, who work part-time, and those with a low education level [23]. Marital status appears it doesn't have any influence in developing depression in these patients [21]. However, few data provide information on the actual role of sociodemographic factors about anxiety. Anxiety levels reported differ significantly by gender. Women are predisposed to react more anxiously in situations that evoke anxiety response [24].

These findings about chronic neck pain and its associated psychological factors urge health care practitioners to consider and identify psychological obstacles to recovery. Understanding the importance of the psychosocial pathway in the incidence and persistence of neck pain lies not only in the advancement of knowledge in the phenomenon, but also in designing preventive interventions, and could contribute in the development of new management strategies in clinical practice.

Even though our study underlines the prevalence of psychic distress in neck pain patients and the relationship between it, functional disability and CBN, it has some limitations linked to sample size, number of exclusions, study type (retrospective) and unmeasured factors of working conditions. Physical job-demand characteristics and ergonomic factors as well as work-related stress can be risk factors and prognostic factors for neck pain.

As depression and anxiety may be caused or aggravated by job demands, working conditions may modify the interaction of psychosocial factors with neck pain. These factors are therefore also to be considered for assessment and management of neck pain.

## Conclusion

This study underlines the fact that both anxiety and depression are prevalent in chronic neck pain (CNP) patients. Furthermore, disability and CBN which are linked to CNP can predict which patient is at higher risk of developing psychological distress. Thus, treatment regimens for neck pain should not only target pain symptoms but should also aim to prevent depressive and anxiety disorders. Nevertheless, further larger studies are needed to assess other risk factors probably related to anxiety and depression in neck pain patients.

### What is known about this topic

- Chronic pain often has a psychic impact;
- Chronic neck pain, as an example, was been associated with psychosocial distress;
- Chronic low back pain can be a cause of anxiety and/or depression. But no study assessed the prevalence of anxiety and depressive disorders in chronic neck pain patients.

### What this study adds

- This study tries to assess the prevalence of anxiety and depression in patients with chronic neck pain and to identify risk factors associated with.
